# Prediction model of artificial neural network for the risk of hyperuricemia incorporating dietary risk factors in a Chinese adult study

**DOI:** 10.29219/fnr.v64.3712

**Published:** 2020-01-20

**Authors:** Jie Zeng, Junguo Zhang, Ziyi Li, Tianwang Li, Guowei Li

**Affiliations:** 1 Center for Clinical Epidemiology and Methodology (CCEM), Guangdong Second Provincial General Hospital, Guangzhou, China; 2 Department of Rheumatology and Immunology, Guangdong Second Provincial General Hospital, Guangzhou, China

**Keywords:** hyperuricemia, dietary factors, artificial neural network, prediction model

## Abstract

**Background:**

Risk of hyperuricemia (HU) has been shown to be strongly associated with dietary factors. However, there is scarce evidence on prediction models incorporating dietary factors to estimate the risk of HU.

**Objective:**

The aim of this study was to develop a prediction model to predict the risk of HU in Chinese adults based on dietary information.

**Design:**

Our study was based on a cross-sectional survey, which recruited 1,488 community residents aged 18 to 60 years in Beijing from October 2010 to January 2011. The eligible participants were randomly divided into a training set (*n*_1_ = 992) and a validation set (*n*_2_ = 496) in the ratio of 2:1. We developed the prediction model in three stages. We first used a logistic regression model (LRM) based on the training set to select a set of dietary risk factors which were related to the risk of HU. Artificial neural network (ANN) was then used to construct the prediction model using the training set. Finally, we used receiver operating characteristic (ROC) curve analysis to assess the accuracy of the prediction model using training and validation sets.

**Results:**

In the training set, the mean age of participants with and without HU was 39.3 (standard deviation [SD]: 9.65) and 38.2 (SD: 9.38) years, respectively. Patients with HU consisted of 101 males (77.7%) and 29 females (22.3%). The LRM found that food frequency (vegetables [odds ratio (OR) = 0.73], meat [0.72], eggs [0.80], plant oil [0.78], tea [0.51], eating habits (breakfast [OR = 1.28]), and the salty cooking style (OR = 1.33) were associated with risk of HU. In the ANN analysis, we selected a three-layer back propagation neural network (BPNN) model with 14, 3, and 1 neuron in the input, hidden, and output layers, respectively, as the best prediction model. The areas under the ROC of the training and validation sets were 0.827 and 0.814, respectively. HU would occur when the incidence probability is greater than 0.128. The indicators of accuracy, sensitivity, specificity, and Yuden Index suggested that the ANN model in our study is successful and valuable.

**Conclusions:**

This study suggests that the ANN model could be used to predict the risk of HU in Chinese adults. Further prospective studies are needed to improve the accuracy and to generalize the use of model.

## Popular scientific summary

- We developed an artificial neural network prediction model to identify the risk of hyperuricemia (HU) in Chinese adults based on dietary risk factors.
- We found that the food frequency of vegetables, meat, eggs, plant oil, tea, eating habits of breakfast and the salty cooking style were associated with the risk of HU.
- The indicators suggested that the study analysis is successful and valuable.
- This study is noninvasive and easier to assess when compared with serum uric acid-related examinations.

Hyperuricemia (HU), which is defined as serum uric acid (SUA) > 420 mmol/L for men, and >360 mmol/L for women (1), is a major cause of disability because of its high prevalence globally (2). In 2011, the prevalence rate of HU ranged from 2.6 to 36% in different populations worldwide (3). In China, with economic development and lifestyle changes, the prevalence of HU has increased rapidly. By the year 2010, the prevalence reached 13.7% (21% in males and 7.9% in females) in northern and northeastern Chinese provinces (4, 5). Emerging evidence has indicated that HU could increase the risk of gout, hypertension, cardiovascular disease, diabetes, and chronic kidney diseases (5, 6).

Epidemiologic evidence has indicated that incidence of HU is strongly related to dietary factors (7, 8). For example, intake of meat, seafood, alcohol, and sugar-sweetened beverages are positively correlated with the risk of HU, while intake of fruits is found to be correlated with the reduced risk of HU (9). However, in research exploring the association between dietary patterns and risk of HU, where the complicated interaction of various groups of foods were considered, the results were conflicting. In China, a cross-sectional study with 374 participants demonstrated that the ‘animal products and fried foods’ dietary pattern was associated with a higher prevalence of HU, while ‘soybean products and fruit’ pattern was associated with its lower prevalence (10). Another cross-sectional study with 266 participants has found that there was no significant association between dietary patterns and uric acid levels (11). Moreover, in previous studies, potential confounders such as age, gender, education, ethnicity, and body mass index (BMI) were not adjusted properly (12). Therefore, it is necessary to identify the association between dietary factors and HU to establish the prediction model.

In the development of HU, the complex combinations of foods interact in non-linear biological mechanisms, which probably need a special mathematical approach, such as artificial neural networks (ANNs) (13), as traditional statistical prediction and classification methods (such as linear regression models [LR], logistic regression models [LRM], etc.) are difficult to solve collinearity problems. Some previous studies have presented evidence that ANN was more powerful than most of the traditional statistical prediction methods (14, 15), but no studies have investigated the ability of ANNs in predicting risk of HU incorporating dietary risk factors in China.

Therefore, the aim of this study is to illustrate the potential usefulness of artificial intelligence, particularly ANN, in predicting the risk of HU vis-à-vis dietary factors. This model can be used as a preliminary screening tool to evaluate associations between HU and dietary risk factors, which would help with health management and prevention of HU in adults.

## Materials and methods

### The subjects

**Fig. 1:**
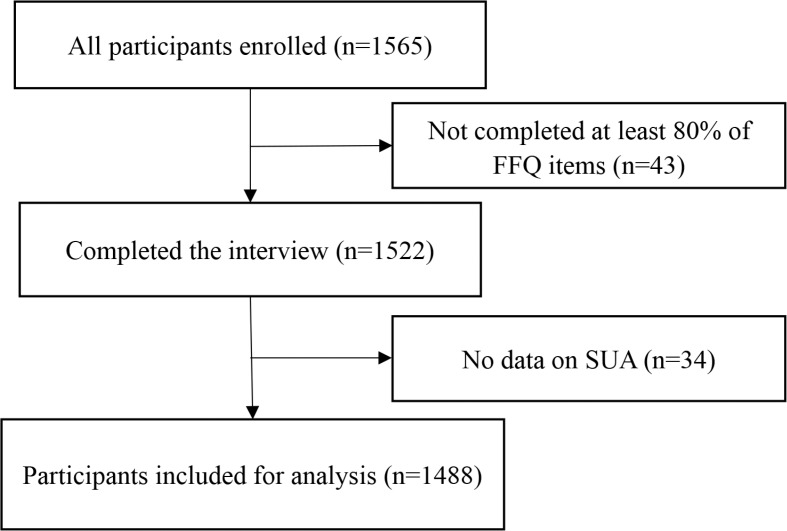
Flow diagram showing selection process of participants in our study.

Our study was based on a cross-sectional study that recruited 1,565 adults aged 18–61 years. The participants were enrolled randomly at a Beijing community hospital according to computer-generated random numbers for health checkup between October 2010 and January 2011. Participants with previously diagnosed gout were excluded. The respondents who did not complete at least 80% of the food frequency questionnaire (FFQ) (*n* = 43) and those having >20% missing SUA measures (*n* = 34) were excluded from analysis. Finally, 1,488 participants were included for analysis. The selection process of participants in this study is shown in Fig. 1.

### Data collection

On the day of recruitment, all subjects had a face-to-face interview with well-trained interviewers using two questionnaires. The questionnaires were designed by experts from our research team and were modified and validated in a pilot study. The first questionnaire included socio-demographic characteristics, smoking, and drinking status. The second questionnaire was a semi-quantitative FFQ. It comprised the following 11 food groups: cereals, fruits, vegetables, meat, seafood, eggs, dairy products, legumes, plant oil, animal oil, and tea, and it was developed based on the Dietary Guidelines for Chinese People (2016) (16). Participants were required to select a category best applicable to them on the basis of past 3 months: consumed rarely, once to three times a month, once a week, twice to four times a week, five to six times a week, once a day, twice or thrice a day, or four times a day or more. The categories were subsequently classified into either ‘low’ group or ‘high’ group according to Dietary Guidelines (16). Additionally, the FFQ contained five questions on eating habits (breakfast frequency, midnight snack frequency, meal time regularity, dining out frequency, and snacking frequency) and three questions on cooking styles (the amount [gram per person per day] of sugar, oil, and salt used when preparing or cooking food). The categories were subsequently classified into either ‘regular’ group or ‘non-regular’ group according to the Dietary Guidelines (16). Serum uric acid levels were measured from venous blood collected in the morning after at least 8 h of fasting. Basic information measurements (weight, height, waist circumference [WC], and blood pressure) were measured by trained interviewers using standard protocol and validated equipment on the day of interview. Body mass index was calculated by the following formula:

BMI (kg/m^2^) = weight/height^2^.

### Statistical analysis

All selected participants (*n* = 1,488) were randomly divided into two sets: training set (*n*_1_ = 992) and validation set (*n*_2_ = 496) in the ratio of 2:1. The process was based on deep learning of ANN for proportional division (17, 18). Differences in participants’ characteristics in the two sets were compared using *x*^2^ test for categorical variables (*t*-test for continuous variables). We used the training set to select variables and establish the predictive ANN model. Then we tested and evaluated the ANN model using the validation set. All variable values were normalized to the range of 0 to 1. The binary variables were divided into 0 and 1, which means ‘No’ and ‘Yes’, respectively. Nonbinary variables were normalized as *X*’_m_ = (*X*_m_ – *X*_min_)/(*X*_max_ – *X*_min_). Continuous variables were reported as mean values (standard deviation [SD]), and categorical variables were expressed as frequency percentages.

The model analysis was divided into three stages. In the first stage, a set of predictors to the HU risk was identified by logistic regression analysis using the training set. Univariate binary LRM was used to evaluate correlation between dietary risk factors and HU. Odds ratios (ORs) and 95% Confidence Intervals (CI) were used to quantify this relationship. In the second stage, we developed an ANN model for predicting HU risk by the input of significant predictors identified in the first stage. The ANN model was initially conducted after choosing potential significant risk factors with a *P*-value < 0.20 to select all possible predictors from univariate logistic models (19). The neural network generally consists of three layers: input layer to receive information, hidden layer to process information, and output layer to calculate response. As for ANN, it is a mathematical model or computational model that attempts to simulate the structure or function of biological neural network (20). Moreover, it is a non-linear statistical data modeling tool that can be used to model complex relationship between input and output. There are different types of neural networks, including feed-forward neural network, radial basis function (RBF) network, and Kohonen self-organizing network (21, 22). The feed-forward neural networks, which include back-propagation (BP) delta rule network and other networks, are the earliest and simplest ANNs, while the BP network is the most popular choice because of its relative simplicity and stability. Therefore, in this study, we used BP for analyses.

In the third stage, we assessed the performance of risk evaluation model (using training and validation set) by using accuracy, sensitivity (Se), specificity (Sp), Yuden Index, and ROC curve analysis to evaluate the model’s discriminatory ability. The accuracy index measures the percentage of correctly diagnosed subjects. Sensitivity refers to the proportion of subjects having target condition and gives positive test results. Specificity is the proportion of subjects without any target condition and gives negative test results. ROC curves display true-positives versus false-positives graphically across a range of cut-offs and of selecting the optimal cut-off for clinical support used. Youden's index is the sum of Se and Sp minus one (Se + Sp – 1) (23). Unless specified, we used the significance level of 0.05 for all analyses. All analyses were performed using the software R version 3.5.3 (R Foundation for Statistical Computing, Vienna, Austria) and the ANN model based on ‘neuralnet’ packages.

## Results

### Description of the participants

Supplementary Table S1 shows that the mean age of participants was over 37 years, with a mean age of 37.7 (9.6) years in the training set and 37.7 (9.8) years in the validation set. There were 521 males and 471 females (47.5%) in the training set, and 256 males and 240 females (48.4%) in the validation set. Differences between the training set and the validation set were not substantial and statistically significant in our study. Participants in these two data sets had similar characteristics (*P* > 0.05).

Table 1 shows that the mean age of participants with HU in the training set was 39.3 (9.7) years and 38.2 (9.4) years in for participants without HU. Participants with HU included 101 males (77.7%) and 29 females (22.3%). We found that the risk factors of gender, age, smoking status, drinking status, SUA, BMI, WC, systolic blood pressure (SBP), diastolic blood pressure (DBP), vegetable frequency, meat frequency, eggs frequency, plant oil frequency, tea frequency, breakfast frequency, and the salty cooking style had statistically significant differences between the participants with and without HU in the training data set (*P* < 0.05).

**Table 1:** Characteristics of participants in training data set by hyperuricemia status

Characteristics	Hyperuricemia		
	Yes (*n* = 130)	No (*n* = 788)	*P-value*
Gender: *n* (%)			
Males	101 (77.7)	382 (48.5)	<0.001^**^
Females	29 (22.3)	406 (51.5)	
Mean age (SD), years	39.33 (9.65)	38.19 (9.38)	0.20*
Ethnicity: *n* (%)			
Han	117 (90.0)	699 (88.7)	0.66
Others	13 (10.0)	89 (11.3)	
Education level: *n* (%)			
High school or under	94 (72.3)	559 (70.9)	0.75
College or higher	36 (27.7)	229 (29.1)	
Smoking status: *n* (%)			
Current smokers	21 (17.1)	97 (13.1)	0.13^*^
Ex-smokers and non-smokers	102 (82.9)	646 (86.9)	
Drinking status: *n* (%)			
Current drinkers	57 (46.3)	264 (35.7)	0.02^*^
Ex-drinkers and non-drinkers	66 (53.7)	476 (64.3)	
Serum uric acid (SUA): mean (SD), μmol/L	454.03 (56.57)	294.21 (61.67)	<0.001^**^
Body mass index (BMI): mean (SD), kg/m^2^	26.37 (3.68)	23.46 (3.10)	<0.001^**^
Waist circumference (WC): mean (SD), cm	88.31 (10.52)	79.36 (9.78)	<0.001^**^
Systolic blood pressure (SBP): mean (SD), mmHg	132.18 (14.86)	121.72 (15.41)	<0.001^**^
Diastolic blood pressure (DBP): mean (SD), mmHg	83.68 (12.10)	75.96 (10.47)	<0.001^**^
*Food frequency*			
Cereals^a^ *n* (%)			
Low	101 (78.3)	609 (79.7)	0.52
High	28 (21.7)	155 (20.3)	
Fruits^b^ *n* (%)			
Low	77 (59.2)	471 (61.2)	0.92
High	53 (40.8)	299 (38.8)	
Vegetables^c^ *n* (%)			
Low	112 (86.8)	649 (84.6)	0.20^*^
High	17 (13.2)	118 (15.4)	
Meat^d^ *n* (%)			
Low	100 (76.9)	538 (70.3)	0.07^*^
High	30 (23.1)	227 (29.7)	
Seafood^e^ *n* (%)			
Low	94 (73.4)	576 (75.5)	0.94
High	34 (26.6)	187 (24.5)	
Eggs^f^ *n* (%)			
Low	90 (70.9)	492 (63.9)	0.18^*^
High	37 (29.1)	278 (36.1)	
Dairy products^g^ *n* (%)			
Low	101 (77.7)	574 (74.4)	0.78
High	29 (22.3)	197 (25.6)	
Legumes^h^ *n* (%)			
Low	92 (71.9)	550 (71.4)	0.40
High	36 (28.1)	220 (28.6)	
Plant oil^i^ *n* (%)			
Low	105 (82.0)	590 (77.3)	0.20^*^
High	23 (18.0)	173 (22.7)	
Animal oil^j^ *n* (%)			
Low	73 (58.9)	416 (56.2)	0.51
High	51 (41.1)	324 (43.8)	
Tea^k^ *n* (%)			
Low	106 (81.5)	499 (65.2)	<0.001^**^
High	24 (18.5)	266 (34.8)	
*Eating habits*			
Breakfast frequency^l^ *n* (%)			
Non-regular	28 (21.5)	218 (27.8)	0.17^*^
Regular	102 (78.5)	567 (72.2)	
Midnight snack frequency^m^ *n* (%)			
Non-regular	66 (51.2)	393 (50.4)	0.95
Regular	63 (48.8)	387 (49.6)	
Meal time regularity *n* (%)			
Non-regular	109 (85.2)	658 (85.2)	0.55
Regular	19 (14.8)	114 (14.8)	
Dining out frequency^n^ *n* (%)			
Non-regular	96 (74.4)	574 (74.1)	0.81
Regular	33 (25.6)	201 (25.9)	
Snacking frequency^o^ *n* (%)			
Non-regular	91 (71.1)	539 (69.8)	0.24
Regular	37 (28.9)	233 (30.2)	
*Cooking styles*			
Sugary^p^ *n* (%)			
Low	97 (80.2)	614 (83.0)	0.24
High	24 (19.8)	126 (17.0)	
Salty^q^ *n* (%)			
Low	81 (64.8)	529 (70.0)	0.09^*^
High	44 (35.2)	227 (30.0)	
Oily^r^ *n* (%)			
Low	105 (85.4)	638 (85.9)	0.46
High	18 (14.6)	105 (14.1)	

* *P* < 0.2, **P < 0.05.

a,b,c,d,i Low = less than or equal to once a day, high = more than once a day.

e,j Low = less than or equal to four times a week, high = more than four times a week.

f,g,h,k Low = less than or equal to six times a week, high = more than six times a week.

l Always = more than five times a week, rarely = less than five times a week.

m,n Always = more than twice a week, rarely = less than twice a week.

o Always = more than six times a week, rarely = less than six times a week.

p,q Low = less than 13 g per person per day, high = more than 13 g per person per day.

r Low = less than 45 g per person per day, high = more than 45 g per person per day.

### Predictors of HU risk

Table 2 shows the predictors of HU risk identified from logistic regression analysis based on the training set (*n*_1_ = 992). From univariate LRM, we found significantly negative relationships between HU risk and the following factors: females (OR = 0.27, 95% CI: 0.18–0.42), age (OR = 1.01, 95% CI: 0.99–1.03), vegetable frequency ([high vs. low], OR = 0.73, 95% CI: 0.45–1.18), meat frequency (OR = 0.72, 95% CI: 0.51–1.03), eggs frequency (OR = 0.80, 95% CI: 0.58–1.11), plant oil frequency (OR = 0.78, 95% CI: 0.53–1.14), tea frequency (OR = 0.51, 95% CI: 0.36–0.74), and breakfast frequency (OR = 1.28, 95% CI: 0.90–1.82). There were also positive relationships between HU risk and these six factors, including current smoking compared with non-smokers (OR = 1.37, 95% CI: 0.82–2.30), current drinking compared with those who never drink alcohol (OR = 1.56, 95% CI: 1.06–2.29), BMI (OR = 1.29, 95% CI: 1.21–1.37), SBP (OR = 1.04, 95% CI: 1.03–1.06), DBP (OR = 1.06, 95% CI: 1.04–1.08), and the salty cooking style (OR = 1.33, 95% CI: 0.96–1.84).

**Table 2:** Analysis of risk factors on hyperuricemia using univariate logistic regression model

Variable	*B*	Standard Error (SE)	Wald *χ*^2^	*P*	Odds ratio (OR) (95% confidence interval [CI])
Female	−1.31	0.22	34.63	<0.001^**^	0.27 (0.18, 0.42)
Age	0.01	0.01	1.64	0.20^*^	1.01 (0.99, 1.03)
Han ethnicity	−0.14	0.31	0.20	0.66	0.87 (0.47, 1.61)
High education	−0.07	0.21	0.10	0.75	0.94 (0.62, 1.41)
Current smoker	0.32	0.26	1.44	0.13^*^	1.37 (0.82, 2.30)
Currently drinking alcohol	0.44	0.20	5.08	0.02^**^	1.56 (1.06, 2.29)
Body mass index (BMI)	0.26	0.03	66.99	<0.001^**^	1.29 (1.21, 1.37)
Systolic blood pressure (SBP)	0.04	0.01	41.54	<0.001^**^	1.04 (1.03, 1.06)
Diastolic blood pressure (DBP)	0.06	0.01	44.26	<0.001^**^	1.06 (1.04, 1.08)
*Food frequency*^a^					
Cereals	−0.13	0.20	0.42	0.52	0.88 (0.59, 1.30)
Fruits	−0.02	0.16	0.01	0.92	0.99 (0.72, 1.35)
Vegetables	−0.32	0.25	1.67	0.2^0*^	0.73 (0.45, 1.18)
Meat	−0.33	0.18	3.31	0.07^*^	0.72 (0.51, 1.03)
Seafood	0.02	0.18	0.01	0.94	1.02 (0.71, 1.45)
Eggs	−0.22	0.17	1.78	0.18^*^	0.80 (0.58, 1.11)
Dairy products	0.05	0.18	0.08	0.78	1.05 (0.74, 1.50)
Legumes	−0.15	0.18	0.70	0.40	0.86 (0.61, 1.22)
Plant oil	−0.26	0.20	1.66	0.20^*^	0.78 (0.53, 1.14)
Animal oil	−0.11	0.16	0.43	0.51	0.90 (0.66, 1.23)
Tea	−0.67	0.19	12.95	<0.001^**^	0.51 (0.36, 0.74)
*Eating habits*^b^					
Breakfast frequency	0.25	0.18	1.92	0.17^*^	1.28 (0.90, 1.82)
Midnight snack frequency	−0.01	0.16	0.004	0.95	0.99 (0.73, 1.34)
Meal time regularity	−0.14	0.23	0.35	0.55	0.87 (0.56, 1.37)
Dining out frequency	−0.43	0.18	0.06	0.81	0.96 (0.67, 1.36)
Snacking frequency	−0.21	0.18	1.41	0.24	0.81 (0.57, 1.15)
*Cooking styles*^c^					
Sugary	0.24	0.20	1.39	0.24	1.27 (0.85, 1.88)
Salty	0.28	0.17	2.87	0.09^*^	1.33 (0.96, 1.84)
Oily	0.16	0.22	0.54	0.46	1.18 (0.76, 1.83)

a,c High versus low, reference = low.

b Regular versus non-regular, reference = regular.

* *P* < 0.2, ^**^ *P* < 0.05.

### Prediction models

**Fig. 2:**
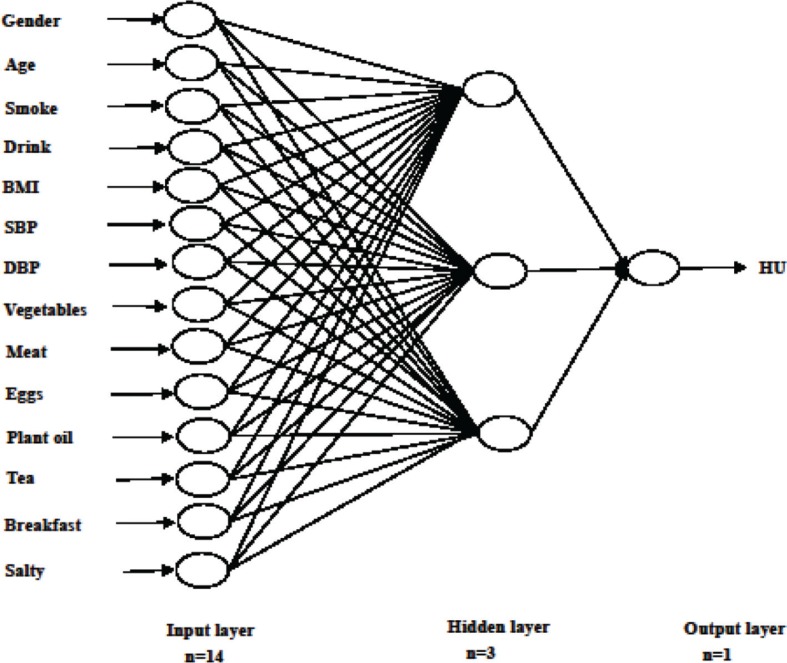
Graphic representation of the basic architecture of artificial network model (ANN) used in the present study for dietary risk analyzed for hyperuricemia in training data set.

We built ANN model on the basis of the predictors of HU risk resulting from logistic regression analysis. The following predictors were used as model inputs: gender, age, smoking status, drinking status, BMI, SBP, DBP, vegetable frequency, meat frequency, eggs frequency, plant oil frequency, tea frequency, breakfast frequency, and the salty cooking style. The binary variable of whether an individual was suffering from HU was the output variable. In this analysis, the structure of BP neural network included three layers (Fig. 2). Parameters were selected according to previous related studies (14, 15). The training parameters such as learning rate and momentum were set at their default values. The training function was based on the Levenberg–Marquardt algorithm. The neural network was trained for 100 epochs. Dropping out 20% of input units and 50% of hidden units was often found to be optimal. It was a simple way to prevent neural networks from over-fitting (24). Each data point was weighed based on its outcome ratio, which was done to ensure that the output result was not heavily skewed toward dominant class. There were 14 neurons in the input layer, three neurons in the hidden layer, and neurons in the output layer of the ANN model corresponding to the forecast variable (that is the probability of having HU).

### Discriminatory ability of models

**Fig. 3:**
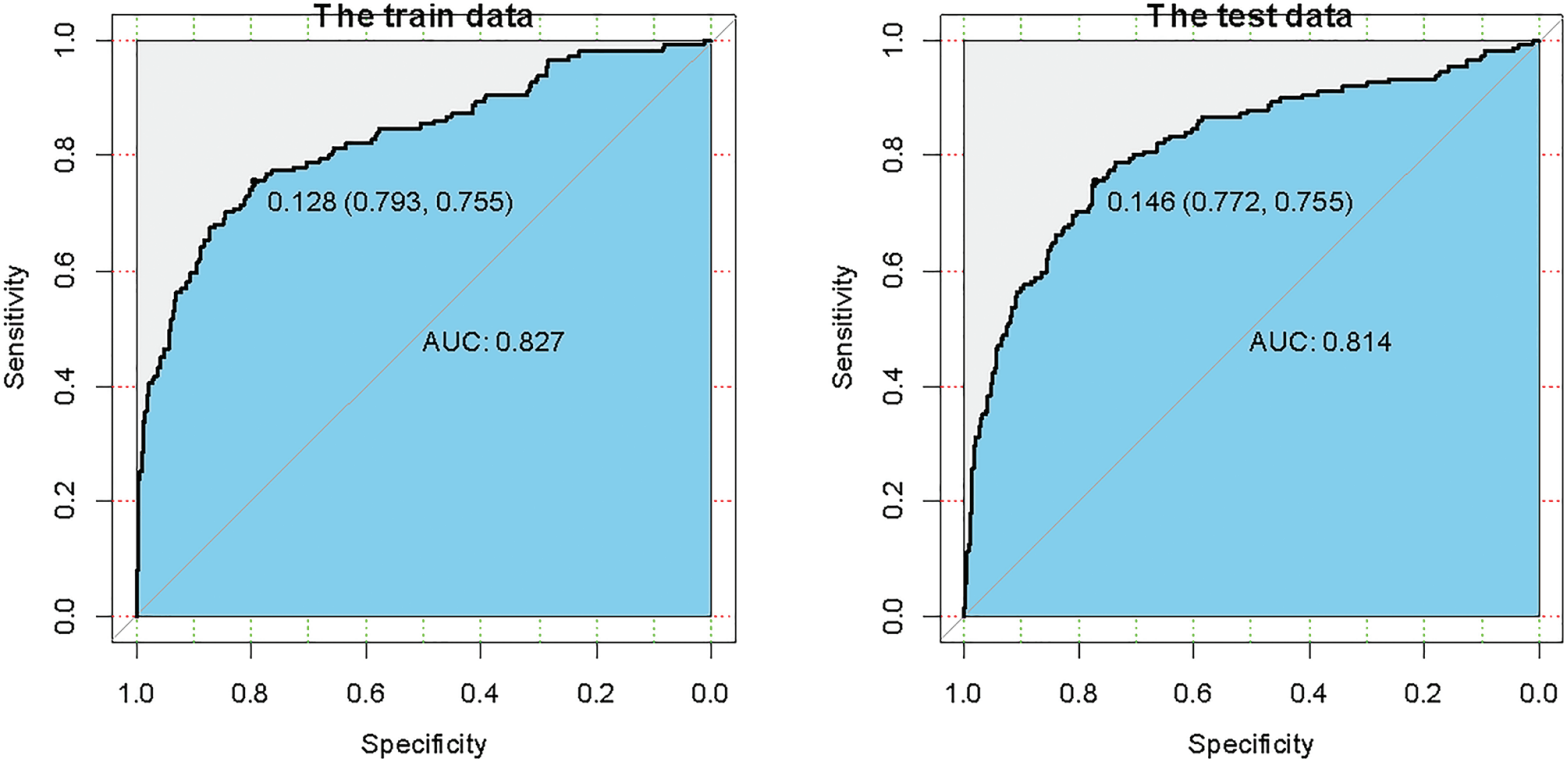
The receiver operating characteristic (ROC) curves obtained from the artificial network model (ANN) in training and test data sets.

Figure 3 summarizes the areas under ROC curves obtained from training and validation sets of ANN model. The area under receiver operating curves (AUCs) was 0.827 for training set and 0.814 for validation set. Thus, a well-trained optimal ANN model here could successfully predict the individual risk of HU, with high accuracy and large AUC. The cut-off incidence probability values of HU were 0.128 for training set and 0.146 for validation set. This means that HU would occur if the incidence probability is greater than 0.128. Table 3 shows that the indicators of accuracy on training and validation sets are 0.84 and 0.80, respectively. The Se, Sp, and Yuden Index on training set (validation set) are 0.75 (0.72), 0.86 (0.83), and 0.70 (0.68), respectively.

**Table 3:** The performance of artificial neural network (ANN) model on training and validation sets

Indicator	Training set (*n* = 992 )	Validation set (*n* = 496)
Accuracy	0.84	0.80
Sensitivity	0.75	0.72
Specificity	0.86	0.83
Yuden index	0.70	0.68
Area under the receiver operating curve (AUC) (95% confidence interval [CI])	0.827 (0.820, 0.832)	0.814 (0.803, 0.824)

## Discussion

This is the first study to develop a HU prediction ANN model based on dietary risk factors because the use of ANN in medical field is a newly emerging phenomenon. In our study, the ANN model was proved to improve the predictive accuracy of HU. In this analysis, we developed a HU prediction model involving 14 significant predictors. For training and validation sets, the AUCs of ANN model were 0.827 and 0.814, respectively. The cut-off incidence probability values of HU were 0.128 for training set and 0.146 for validation set. Our study found that HU would occur when the incidence probability is greater than 0.128. Furthermore, we did a *post-hoc* analysis and found that the AUCs based on ANN and LRM were 0.827 and 0.758, respectively (*P* < 0.05). A previous study by Jahandideh et al. (25) found that ANN model was a better technique to predict the presence of coronary artery disease than LRM, which was similar to our study. More importantly, the indicators of accuracy, Se, Sp, and Yuden Index suggested that the ANN model in our study is successful and valuable. ANN is a systematic tool with great potential for clinical decision support; it could establish specific prediction value for each patient according to their related risk factors. Ability to provide targeted prediction is the most obvious strength of ANN comparing with the traditional statistical analysis methods.

The application of ANN model is of great significance in public health. It could be used as a preliminary screening tool to identify individual at high risk of HU based on their dietary factors; it could also guide the prevention strategy in clinic. The predictors included in the model are common and readily available, and could be assessed easily without any invasion. Moreover, this model could be applied to general population as well. New models’ programs could be easily uploaded in computers. Therefore, if we put 14 dietary predictors in a program, the computer would automatically calculate the risk probability of HU. Owing to its simplicity, this could be more efficient than those traditional diagnostic techniques, which are more expensive and complex. However, whether the forecast probability could be applied to a particular individual requires further exploration.

Our study has found that dietary factors are major predictors of HU. Positive relationships were found between HU and the following factors: current smoking, current drinking, BMI, SBP, DBP, and the salty cooking style, while the negative relationships were found between HU risk and the following factors: female, age, vegetable frequency, meat frequency, eggs frequency, plant oil frequency, tea frequency, and breakfast frequency. In our analysis, higher tea frequency as an independent factor was negatively associated with decreased risk of HU, which was consistent with a Chinese epidemiologic study (26). The explanation could be that the caffeine found in tea could protect against increasing SUA because of its diuretic and antioxidative properties (27, 28). However, meat and egg intake was positively associated with the prevalence of HU, which was not consistent with a previous Chinese study (8). This may vary with different regions and populations.

Some studies have indicated that HU was affected prominently by lifestyles such as diet habits and demographic characteristics (29–32). For instance, some previous studies have found that high intake of salt was strongly related to HU (33, 34). Several metabolic experiments in both animals and humans have proved the effect of high loading of purine on increased serum urate level (31, 35, 36). Moreover, people who skip breakfast will be hungry and eat a lot of foods rich in protein and purines for lunch, which would accelerate the occurrence of HU (37). The habit of smoking would increase carbon monoxide in the blood-forming carboxyhemoglobin, which could induce erythrocytosis because of inadequate oxygenation in blood circulation (38). Increased total red blood cell count may lead to a large amount of red blood cells being destroyed, which would promote purine metabolism and overproduction of uric acid (39).

Our study has several strengths. First, the data are of high quality because these were collected by well-trained investigators using validated questionnaires and equipment. Second, our study used ANN model based on dietary factors to predict the incidence of HU, which is a novel method seldom used in previous studies. The accuracy of the prediction was improved by using ANN, compared with traditional prediction models. Third, the model could be used to predict HU risk by dietary factors, which is non-invasive and easier to assess compared with SUA examination. It could potentially improve the prevention of HU, especially in poor areas where the medical service is insufficient.

There are some limitations to our study. First, the samples were limited geographically and ethnically, so the generalization of the results should be taken with caution. Second, as a cross-sectional survey, the study was unable to verify the causality or the temporal relationship between diet and HU. Third, there may be some potential bias included in the models that would influence our findings. Furthermore, ANN models may be less practical to be used in clinic, as it is more complex than LRM and LR models (40, 41), leading to a higher requirement of statistical background of researcher. Last but not least, FFQ may introduce recall bias inevitably, which would result in non-differential misclassification, leading to association toward null (42). Despite these limitations, we developed an accurate risk predictive model that estimates the combined impact of an individual’s dietary factors on HU.

## Conclusions

In conclusion, our study found that food frequency, eating habits, and cooking styles were associated with HU risk in Chinese adults. The results showed that ANN model could be used to improve the predictive accuracy of HU. HU would occur if the incidence probability is greater than 0.128. Further prospective studies are needed to confirm the findings and to validate our model for predicting HU risk in adults.

## Supplementary Material

Prediction model of artificial neural network for the risk of hyperuricemia incorporating dietary risk factors in a Chinese adult study

## References

[1] Liu L, Lou S, Xu K, Meng Z, Zhang Q, Song K Relationship between lifestyle choices and hyperuricemia in Chinese men and women. Clin Rheumatol 2013; 32: 233–9. 10.1007/s10067-012-2108-z 23132661

[2] Liu H, Zhang XM, Wang YL, Liu BC Prevalence of hyperuricemia among Chinese adults: a national cross-sectional survey using multistage, stratified sampling. J Nephrol 2014; 27: 653–8. 10.1007/s40620-014-0082-z 24687401

[3] Uaratanawong S, Suraamornkul S, Angkeaw S, Uaratanawong R Prevalence of hyperuricemia in Bangkok population. Clin Rheumatol 2011; 30: 887–93. 10.1007/s10067-011-1699-0 21302126

[4] Trifiro G, Morabito P, Cavagna L, Ferrajolo C, Pecchioli S, Simonetti M, et al. Epidemiology of gout and hyperuricaemia in Italy during the years 2005–2009: a nationwide population-based study. Ann Rheum Dis 2013; 72: 694–700. 10.1136/annrheumdis-2011-201254 22736095

[5] Chen S, Guo X, Dong S, Yu S, Chen Y, Zhang N, et al. Association between the hypertriglyceridemic waist phenotype and hyperuricemia: a cross-sectional study. Clin Rheumatol 2017; 36: 1111–19. 10.1007/s10067-017-3559-z 28185015

[6] Wang J, Qin T, Chen J, Li Y, Wang L, Huang H, et al. Hyperuricemia and risk of incident hypertension: a systematic review and meta-analysis of observational studies. PLoS One 2014; 9: e114259 10.1371/journal.pone.0114259 25437867 PMC4250178

[7] Liu R, Han C, Wu D, Xia X, Gu J, Guan H, et al. Prevalence of hyperuricemia and gout in mainland China from 2000 to 2014: a systematic review and meta-analysis. BioMed Res Int 2015; 2015: 762820 10.1155/2015/762820 26640795 PMC4657091

[8] Liu X, Huang S, Xu W, Zhou A, Li H, Zhang R, et al. Association of dietary patterns and hyperuricemia: a cross-sectional study of the Yi ethnic group in China. Food Nutr Res 2018; 62 10.29219/fnr.v62.1380 PMC5917417 29720927

[9] Gao X, Qi L, Qiao N, Choi HK, Curhan G, Tucker KL, et al. Intake of added sugar and sugar-sweetened drink and serum uric acid concentration in US men and women. Hypertension 2007; 50: 306–12. 10.1161/HYPERTENSIONAHA.107.091041 17592072

[10] Zhang M, Chang H, Gao Y, Wang X, Xu W, Liu D, et al. Major dietary patterns and risk of asymptomatic hyperuricemia in Chinese adults. J Nutr Sci Vitaminol 2012; 58: 339–45. 10.3177/jnsv.58.339 23327969

[11] Tsai YT, Liu JP, Tu YK, Lee MS, Chen PR, Hsu HC, et al. Relationship between dietary patterns and serum uric acid concentrations among ethnic Chinese adults in Taiwan. Asia Pac J Clin Nutr 2012; 21: 263–70. PMID: . 22507614

[12] Yu KH, See LC, Huang YC, Yang CH, Sun JH Dietary factors associated with hyperuricemia in adults. Semin Arthritis Rheum 2008; 37: 243–50. 10.1016/j.semarthrit.2007.04.007 17570471

[13] Eller-Vainicher C, Zhukouskaya VV, Tolkachev YV, Koritko SS, Cairoli E, Grossi E, et al. Low bone mineral density and its predictors in type 1 diabetic patients evaluated by the classic statistics and artificial neural network analysis. Diabetes Care 2011; 34: 2186–91. 10.2337/dc11-0764 21852680 PMC3177712

[14] Disse E, Ledoux S, Betry C, Caussy C, Maitrepierre C, Coupaye M, et al. An artificial neural network to predict resting energy expenditure in obesity. Clin Nutr 2018; 37: 1661–9. 10.1016/j.clnu.2017.07.017 28893410

[15] Shaabanpour Aghamaleki F, Mollashahi B, Nosrati M, Moradi A, Sheikhpour M, Movafagh A Application of an artificial neural network in the diagnosis of chronic lymphocytic leukemia. Cureus 2019; 11: e4004 10.7759/cureus.4004 31001458 PMC6450593

[16] Wang SS, Lay S, Yu HN, Shen SR Dietary guidelines for Chinese residents (2016): comments and comparisons. J Zhejiang Univ Sci B 2016; 17: 649–56. 10.1631/jzus.B1600341 27604857 PMC5018612

[17] Hearty AP, Gibney MJ Analysis of meal patterns with the use of supervised data mining techniques – artificial neural networks and decision trees. Am J Clin Nutr 2008; 88: 1632–42. 10.3945/ajcn.2008.26619 19064525

[18] Gupta H, Gupta PK, Fang X, Miller WJ, Cemaj S, Forse RA, et al. Development and validation of a risk calculator predicting postoperative respiratory failure. Chest 2011; 140: 1207–15. 10.1378/chest.11-0466 21757571

[19] Miles J SM Appling regression and correlation: a guide for students. London: Sage; 2001.

[20] Mukamal KJ, Ding EL, Djousse L Alcohol consumption, physical activity, and chronic disease risk factors: a population-based cross-sectional survey. BMC Public Health 2006; 6: 118 10.1186/1471-2458-6-118 16670030 PMC1475847

[21] Montie JE, Wei JT Artificial neural networks for prostate carcinoma risk assessment. An overview. Cancer 2001; 91: 1647–52. 10.1002/1097-0142 11309763

[22] Grossi E How artificial intelligence tools can be used to assess individual patient risk in cardiovascular disease: problems with the current methods. BMC Cardiovasc Disord 2006; 6: 20 10.1186/1471-2261-6-20 16672045 PMC1479368

[23] Florkowski CM Sensitivity, specificity, receiver-operating characteristic (ROC) curves and likelihood ratios: communicating the performance of diagnostic tests. Clin Biochem Rev 2008; 29(Suppl 1): S83–S87. PMCID: . 18852864 PMC2556590

[24] Defernez M, Kemsley EK Avoiding overfitting in the analysis of high-dimensional data with artificial neural networks (ANNs). Analyst 1999; 124: 1675–81. 10.1039/a905556h 26114398

[25] Jahandideh S, Abdolmaleki P, Movahedi MM Comparing performances of logistic regression and neural networks for predicting melatonin excretion patterns in the rat exposed to ELF magnetic fields. Bioelectromagnetics 2010; 31: 164–71. 10.1002/bem.20541 19771546

[26] Yu JW, Yang TG, Diao WX, Cai XQ, Li T, Zhong H, et al. Epidemiological study on hyperuricemia and gout in Foshan areas, Guangdong province. Zhonghua Liu Xing Bing Xue Za Zhi 2010; 31: 860–2. PMID: . 21162982

[27] Pham NM, Yoshida D, Morita M, Yin G, Toyomura K, Ohnaka K, et al. The relation of coffee consumption to serum uric acid in Japanese men and women aged 49–76 years. J Nutr Metab 2010; 2010 10.1155/2010/930757 PMC2925214 20798877

[28] Donmez C, Konac E Might E-cadherin promoter polymorphisms of rs16260 and rs5030625 associate with the risk of nephrolithiasis? Springerplus 2016; 5: 1673 10.1186/s40064-016-3363-2 27733975 PMC5040654

[29] Beydoun MA, Canas JA, Fanelli-Kuczmarski MT, Tajuddin SM, Evans MK, Zonderman AB Genetic risk scores, sex and dietary factors interact to alter serum uric acid trajectory among African-American urban adults. Br J Nutr 2017; 117: 686–97. 10.1017/S0007114517000411 28345493 PMC5679207

[30] Li R, Yu K, Li C Dietary factors and risk of gout and hyperuricemia: a meta-analysis and systematic review. Asia Pacific J Clin Nutr 2018; 27: 1344–56. 10.6133/apjcn.201811_27(6).0022 30485934

[31] Xia Y, Xiang Q, Gu Y, Jia S, Zhang Q, Liu L, et al. A dietary pattern rich in animal organ, seafood and processed meat products is associated with newly diagnosed hyperuricaemia in Chinese adults: a propensity score-matched case-control study. Br J Nutr 2018; 119: 1177–84. 10.1017/S0007114518000867 29759111

[32] Zhang Y, Liu Y, Qiu H Association between dietary zinc intake and hyperuricemia among adults in the United States. Nutrients 2018; 10: 568 10.3390/nu10050568 PMC5986448 29734733

[33] He FJ, Burnier M, Macgregor GA Nutrition in cardiovascular disease: salt in hypertension and heart failure. Eur Heart J 2011; 32: 3073–80. 10.1093/eurheartj/ehr194 21705359

[34] Liu CW, Chang WC, Lee CC, Chen KH, Wu YW, Hwang JJ Hyperuricemia is associated with a higher prevalence of metabolic syndrome in military individuals. Mil Med 2018; 183: e391–95. 10.1093/milmed/usy097 29750266

[35] Kou Y, Li Y, Ma H, Li W, Li R, Dang Z Uric acid lowering effect of Tibetan medicine RuPeng15 powder in animal models of hyperuricemia. J Tradit Chin Med 2016; 36: 205–10. 10.1016/s0254-6272(16)30028-0 27400475

[36] Zhang Y, Jin L, Liu J, Wang W, Yu H, Li J, et al. Effect and mechanism of dioscin from Dioscorea spongiosa on uric acid excretion in animal model of hyperuricemia. J Ethnopharmacol 2018; 214: 29–36. 10.1016/j.jep.2017.12.004 29233733

[37] Deshmukh-Taskar PR, Nicklas TA, O'Neil CE, Keast DR, Radcliffe JD, Cho S The relationship of breakfast skipping and type of breakfast consumption with nutrient intake and weight status in children and adolescents: the National Health and Nutrition Examination Survey 1999–2006. J Am Diet Assoc 2010; 110: 869–78. 10.1016/j.jada.2010.03.023 20497776

[38] El-Zayadi AR, Selim O, Hamdy H, El-Tawil A, Moustafa H Heavy cigarette smoking induces hypoxic polycythemia (erythrocytosis) and hyperuricemia in chronic hepatitis C patients with reversal of clinical symptoms and laboratory parameters with therapeutic phlebotomy. Am J Gastroenterol 2002; 97: 1264–65. 10.1111/j.1572-0241.2002.05718.x 12014742

[39] Vaya A, Rivera L, Hernandez-Mijares A, Bautista D, Sola E, Romagnoli M, et al. Association of metabolic syndrome and its components with hyperuricemia in a Mediterranean population. Clin Hemorheol Microcirc 2015; 60: 327–34. 10.3233/CH-141887 25261431

[40] Harrison RF, Kennedy RL Artificial neural network models for prediction of acute coronary syndromes using clinical data from the time of presentation. Ann Emerg Med 2005; 46: 431–39. 10.1016/j.annemergmed.2004.09.012 16271675

[41] Ozsen S, Kara S, Latifoglu F, Gunes S A new supervised classification algorithm in artificial immune systems with its application to carotid artery Doppler signals to diagnose atherosclerosis. Comput Methods Programs Biomed 2007; 88: 246–55. 10.1016/j.cmpb.2007.09.002 17976855

[42] Shahar D, Shai I, Vardi H, Brener-Azrad A, Fraser D Development of a semi-quantitative food frequency questionnaire (FFQ) to assess dietary intake of multiethnic populations. Eur J Epidemiol 2003; 18: 855–61. 10.1023/a:1025634020718 14561044

