# Supplementary material for: Prediction model of artificial neural network for the risk of hyperuricemia incorporating dietary risk factors in a Chinese adult study
Source: Food Nutr Res. 2020 Jan 20;64:10.29219/fnr.v64.3712. doi: 10.29219/fnr.v64.3712 (PMC6983978; doi:10.29219/fnr.v64.3712)
Supplement: Prediction model of artificial neural network for the risk of hyperuricemia incorporating dietary risk factors in a Chinese adult study [file FNR-64-3712-s001.doc]

**Supplemental Table 1.** Characteristics of the participants in training set and validation set

| **Characteristic** | **Training set (n=992 )** | **Validation set (n=496 )** | ***P-*value** |
| --- | --- | --- | --- |
| Gender: n(%) |  |  | 0.78 |
| Males | 521 (52.5) | 256 (51.6) |  |
| Females | 471(47.5) | 240 (48.4) |  |
| Mean age(s.d.), years | 37.7 (9.6) | 37.8 (9.8) | 0.19 |
| Ethnicity: *n*(%) |  |  | 0.13 |
| Han nationality | 879 (88.6) | 425 (85.7) |  |
| Others | 113 (11.4) | 71 (14.3) |  |
| Education level: *n*(%) |  |  | 0.95 |
| High school or less | 285 (28.7) | 141 (28.4) |  |
| College or more | 707 (71.3) | 355 (71.6) |  |
| Smoking status: *n*(%) |  |  | 0.74 |
| Current smokers | 132 (14.1) | 68 (14.9) |  |
| Ex- and non-smokers | 805 (85.9) | 388 (85.1) |  |
| Drinking status: *n*(%) |  |  | 0.73 |
| Current drinkers | 352 (37.7) | 178 (38.9) |  |
| Ex- and non-drinkers | 581 (62.3) | 280 (61.1) |  |
| SUA: mean (SD), μmol/L | 316.9 (82.6) | 316.3 (80.6) | 0.82 |
| BMI: mean (SD), kg/m^2^ | 23.8 (3.3) | 23.9 (3.1) | 0.38 |
| WC: mean (SD), cm | 80.3 (10.3) | 80.9 (9.9) | 0.24 |
| SBP: mean (SD), mmHg | 123.2 (15.7) | 122.2 (15.6) | 0.92 |
| DBP: mean (SD), mmHg | 76.9 (11.0) | 77.0 (10.9) | 0.92 |
| ***Food frequency group*** |  |  |  |
| Cereals^a^: *n*(%) |  |  |  |
| Low | 764 (79.4) | 394 (81.6) | 0.33 |
| High | 198 (20.6) | 89 (18.4) |  |
| Fruits^b^: *n*(%) |  |  |  |
| Low | 579 (59.5) | 300 (62.5) | 0.27 |
| High | 394 (40.5) | 180 (37.5) |  |
| Vegetables^c^: *n*(%) |  |  |  |
| Low | 819 (84.6) | 420 (87.7) | 0.12 |
| High | 149 (15.4) | 59 (12.3) |  |
| Meat^d^: *n*(%) |  |  |  |
| Low | 689 (71.2) | 328 (68.0) | 0.22 |
| High | 279 (28.8) | 154 (32.0) |  |
| Seafood^e^: *n*(%) |  |  |  |
| Low | 715 (74.4) | 367 (76.1) | 0.47 |
| High | 246 (25.6) | 115 (23.9) |  |
| Eggs^f^: *n*(%) |  |  |  |
| Low | 633 (65.5) | 305 (63.3) | 0.41 |
| High | 334 (34.5) | 177 (36.7) |  |
| Dairy products^g^: *n*(%) |  |  |  |
| Low | 713 (73.4) | 376 (77.8) | 0.06 |
| High | 259 (26.6) | 107 (22.2) |  |
| Legumes^h^: *n*(%) |  |  |  |
| Low | 689 (71.0) | 331 (68.5) | 0.33 |
| High | 281 (29.0) | 152 (31.5) |  |
| Plant oil^i^: *n*(%) |  |  |  |
| Low | 748 (77.9) | 366 (75.8) | 0.36 |
| High | 212 (22.1) | 117 (24.2) |  |
| Animal oil^j^: *n*(%) |  |  |  |
| Low | 533 (57.0) | 263 (56.3) | 0.81 |
| High | 402 (43.0) | 204 (43.7) |  |
| Tea^k^: *n*(%) |  |  |  |
| Low | 639 (66.1) | 310 (64.7) | 0.61 |
| High | 328 (33.9) | 169 (35.3) |  |
| ***Eating habit*** |  |  |  |
| Breakfast frequency^l^ : *n*(%) |  |  |  |
| Non-Regular | 275 (27.8) | 153 (31.3) | 0.17 |
| Regular | 713 (72.2) | 336 (68.7) |  |
| Midnight snack frequency^m^: *n*(%) |  |  |  |
| Non-Regular | 495 (50.4) | 255 (52.0) | 0.54 |
| Regular | 488 (49.6) | 235 (48.0) |  |
| Meal time regularity: *n*(%) |  |  |  |
| Non-Regular | 831 (85.4) | 419 (86.4) | 0.61 |
| Regular | 142 (14.6) | 66 (13.6) |  |
| Dining out frequency^n^: *n*(%) |  |  |  |
| Non-Regular | 730 (74.6) | 366 (75.5) | 0.73 |
| Regular | 248 (25.4) | 119 (24.5) |  |
| Snacking frequency^o^: *n*(%) |  |  |  |
| Non-Regular | 687 (70.6) | 351 (71.9) | 0.60 |
| Regular | 286 (29.4) | 137 (28.1) |  |
| ***Cooking style*** |  |  |  |
| Sugary^p^: *n*(%) |  |  |  |
| Low | 767 (82.7) | 387 (83.6) | 0.66 |
| High | 161 (17.3) | 76 (16.4) |  |
| Salty^q^: *n*(%) |  |  |  |
| Low | 663 (69.9) | 346 (73.2) | 0.20 |
| High | 286 (30.1) | 127 (26.8) |  |
| Oily^r^: *n*(%) |  |  |  |
| Low | 795 (85.2) | 402 (88.0) | 0.16 |
| High | 138 (14.8) | 55 (12.0) |  |

**Note**: *BMI* body mass index, *WC* waist circumferences, *SBP* systolic blood pressure, *DBP* diastolic blood pressure, *SUA* serum uric acid

^a b^ Low = less than or equal to 6 times a week and once a day, high = more than once a day

^c d^ Low = less than or equal to once a day, high = more than once a day

^e^ Low = less than or equal to 4 times a week, high = more than 4 times a week

^f^ Low = less than or equal to 6 times a week, high = more than 6 times a week

^g h^ Low = less than or equal to 6 times a week, high = more than 6 times a week

^i^ Low = less than or equal to once a day, high = more than once a day

^j^ Low = less than or equal to 4 times a week, high = more than 4 times a week

^k^ Low = less than or equal to 6 times a week, high = more than 6 times a week

^l^ Always = more than 5 times a week, rarely = less than 5 times a week

^m n^ Always = more than 2 times a week, rarely = less than 2 times a week

^o^ Always = more than 6 times a week, rarely = less than 6 times a week

^p^ Low = less than 13 grams per person per day, high = more than 13 grams per person per day

^q^ Low = less than 13 grams per person per day, high = more than 13 grams per person per day

^r^ Low = less than 45 grams per person per day, high = more than 45 grams per person per day
